# The Role of Intravitreal Corticosteroids in the Treatment of DME: Predictive OCT Biomarkers

**DOI:** 10.3390/ijms23147585

**Published:** 2022-07-08

**Authors:** Marion R. Munk, Gabor Mark Somfai, Marc D. de Smet, Guy Donati, Marcel N. Menke, Justus G. Garweg, Lala Ceklic

**Affiliations:** 1 Department of Ophthalmology, Inselspital, Bern University Hospital, University of Bern, 3010 Bern, Switzerland; lalcek@hotmail.com; 2 Bern Photographic Reading Center, Inselspital, University Hospital Bern, 3010 Bern, Switzerland; 3 Department of Ophthalmology, Feinberg School of Medicine, Northwestern University, Chicago, IL 60208, USA; 4 Department of Ophthalmology, Stadtspital Zürich, 8063 Zurich, Switzerland; somfaigm@yahoo.com; 5 Spross Research Institute, 8063 Zurich, Switzerland; 6 Department of Ophthalmology, Faculty of Medicine, Semmelweis University, 1085 Budapest, Hungary; 7 Medical/Surgical Retina and Ocular Inflammation, University of Lausanne, MIOS SA, 1015 Lausanne, Switzerland; mddesmet1@mac.com; 8 Centre Ophtalmologique de la Colline, University of Geneve, 1205 Geneve, Switzerland; guy.donati@bluewin.ch; 9 Department of Ophthalmology, Cantonal Hospital Aarau, 5001 Aarau, Switzerland; marcel.menke@ksa.ch; 10 Swiss Eye Institute, Berner Augenklinik am Lindenhofspital, 3012 Bern, Switzerland; justus.garweg@augenklinik-bern.ch

**Keywords:** diabetes mellitus, diabetic maculopathy, diabetic macular edema, DME, biomarker, OCT, response to treatment, dexamethasone, corticosteroid, anti-VEGF

## Abstract

This work aims to summarize predictive biomarkers to guide treatment choice in DME. Intravitreal anti-VEGF is considered the gold standard treatment for centers involving DME, while intravitreal steroid treatment has been established as a second-line treatment in DME. However, more than 1/3 of the patients do not adequately respond to anti-VEGF treatment despite up to 4-weekly injections. Not surprisingly, insufficient response to anti-VEGF therapy has been linked to low-normal VEGF levels in the serum and aqueous humor. These patients may well benefit from an early switch to intravitreal steroid treatment. In these patients, morphological biomarkers visible in OCT may predict treatment response and guide treatment decisions. Namely, the presence of a large amount of retinal and choroidal hyperreflective foci, disruption of the outer retinal layers and other signs of chronicity such as intraretinal cysts extending into the outer retina and a lower choroidal vascular index are all signs suggestive of a favorable treatment response of steroids compared to anti-VEGF. This paper summarizes predictive biomarkers in DME in order to assist individual treatment decisions in DME. These markers will help to identify DME patients who may benefit from primary dexamethasone treatment or an early switch.

## 1. Introduction

Diabetes mellitus (DM) is a serious and growing healthcare concern worldwide. In the United States of America, 30 million individuals have manifest DM, and a further 86 million are prediabetic [1,2]. Medical costs for persons with DM are twice as high as those of individuals without DM [2]. Diabetic macular edema (DME) is the most common cause of visual disability in the working-age population worldwide [2]. The global prevalence of DME in type 1 and 2 diabetes mellitus (DM) is 11.4% in European countries and 45.3% in North American countries [1]. The Wisconsin Epidemiologic Study of Diabetic Retinopathy found that 20% of patients with DM type 1 and 25% of those with type 2 will develop DME within 10 years after diagnosis [3]. Untreated, 50% of patients with DME lose two or more lines of visual acuity within two years [3].

DME can occur at any stage of diabetic retinopathy but typically in mild-to-moderate diabetic retinopathy (DR) [1]. It is characterized by intra- and subretinal fluid accumulation and retinal thickening of the macula. In both DR and DME, chronic hyperglycemia causes an upregulation of vascular endothelial factor (VEGF), which leads to increased vascular permeability and angiogenesis [1,4,5,6]. Inflammatory mediators play an important role in the pathophysiology of DME and contribute to vascular permeability and edema [7,8,9,10,11,12,13]. The first-line treatments are anti-VEGF drugs, such as aflibercept or ranibizumab, while intravitreal steroids, such as dexamethasone or fluocinolone acetonide implants, are merely used as a second-line treatment. Further therapeutic options include in selected cases, laser therapy and pars plana vitrectomy. Clinical trials such as the RISE/RIDE and VISTA/VIVID pivotal trials showed that 31–46% of patients improved functionally by ≥3 ETDRS letter lines under the anti-VEGF treatment while untreated or laser-treated patients barely showed any improvement [13,14,15,16,17]. In protocol T, 40% had persistent DME after 24 weeks of intensive anti-VEGF treatment [18,19,20]. The reason for this heterogenous response is not yet fully understood. Evidently, DME is a multifactorial and complex disease driven by hypoxia, inflammation, hyperpermeability and angiogenesis. Extracellular as well as intracellular fluid accumulation can occur in DME. However, not all extracellular fluid is vasogenic, as there is re-oriented fluid accumulated through Mueller cells as a result of inflammation and altered expression of aquaporins. 

Studies have shown that especially DME patients with low to normal VEGF levels and higher levels of inflammatory markers, e.g., IL-8, IL-6, IL-1b and ICAM-1 in the serum as well as in the anterior chamber, do not adequately respond to the gold standard anti-VEGF treatment [21,22]. Long-standing, chronic DME, in general, seems to show a limited response to antiangiogenetic drugs [18,23]. Patients insufficiently responsive to anti-VEGF drugs may therefore benefit from the treatment controlling the significant role of inflammation in DME. Based on the OCT examination, several biomarkers have been identified to predict the responsiveness to treatment. These biomarkers can be used to guide individual treatment decisions and an early switch to anti-inflammatory therapy. This review summarizes the most important biomarkers in DME supporting an individualized treatment choice to optimize the outcomes.

## 2. Method

A PubMed and Google Scholar literature search for English language articles was performed using the terms “Diabetic macular edema” AND “Treatment” OR “Diagnosis” OR “Biomarkers” OR “Management” OR “Outcomes”. References cited in selected articles were also reviewed to identify additional relevant reports. Likewise, published national and international guidelines reviews, cases and case series, meta-analyses, and retrospective studies were also scrutinized. References used in this article were discussed upon panel and consensus for relevance. An initial narrative was drafted and reviewed by all authors, who had the opportunity to include changes, suggestions and comments. Based on the panel feedback, the manuscript was finalized. The consensus was achieved upon detailed review, panel discussion, and agreement of all authors. Discrepancies were resolved with discussion and a detailed reference search to prove the level of evidence-based statement.

## 3. General Considerations

**Scheme 1:**
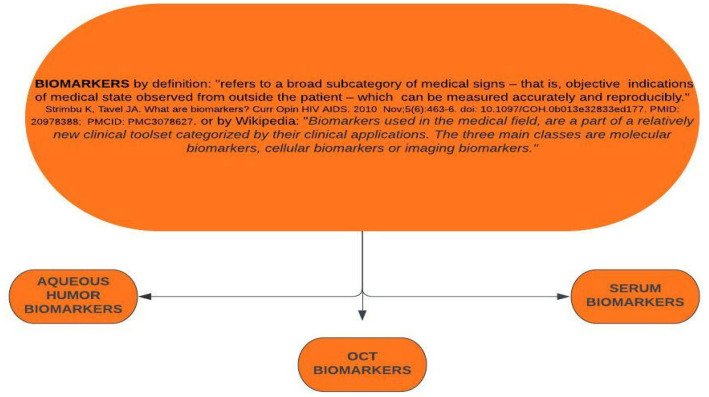
Definitions and subtypes of biomarkers.

The terms and meanings of “predictive” and “prognostic” must be carefully differentiated when biomarkers are described and assessed, particularly as they are often used interchangeably in the medical literature. A prognostic biomarker is a parameter that provides information on the likely patient outcome (e.g., disease recurrence) irrespective of the treatment. A predictive biomarker, in contrast, can identify individuals who are more likely to respond to exposure to a particular medical product or environmental agent. Since predictive biomarkers may guide treatment decisions, we will focus mainly on these markers (Scheme 1). Most of the findings reported here originate from smaller studies, which used conventional statistics and included a limited number of patients, limiting the current state of knowledge. While more advanced statistical approaches such as artificial intelligence (AI) and machine learning (ML) enable us to test for >1000 biomarkers at once, smaller studies using conventional statistics only allow us to test for a couple. 

These conventional analyses might be able to identify single biomarkers, but they do not enable us to see the whole picture, thus the interaction of different biomarkers and their weighed importance. The reason why a patient may be a good responder to first-line therapy or a poor responder is based on the presence (or absence) and complex interaction of a great quantity and variety of parameters, while we here discuss and assess biomarker by biomarker in a univariate way, rather the interaction of all of them lead to the different response profiles of our patients. 

We also focus on morphological biomarkers, which exclude other important factors such as general disease control, duration, and systemic factors, which as well impact the severity and treatment response to a significant degree. Beyond that, we have to be aware that many biomarkers have remained unidentified. This is also highlighted by a recent paper that used ML to assess the prognosis of DME. The model included 312 features, and nonetheless, the R2 was only 0.21. This means that only 21% of the variation in the output variable is actually explained by the input variable(s) [24]. Until we have large-scale data that allow for the prediction of the treatment response of different drugs by AI, we will only have a limited understanding. This may also explain the discrepant and sometimes conflicting findings of different studies discussed here. From a clinical perspective, on the other hand, the following biomarkers may be simple to use in the routine of following patients and their responses to treatment.

## 4. Definition of Poor Response and Persistent DME

Anti-VEGF therapy is the first-line treatment for DME. Other therapeutic options are usually evaluated in case of limited treatment response to the respective drugs. If the macula is persistently thickened and there is a limited functional response, DME is considered refractory to therapy, and the patient is classified as a poor responder. However, the exact definition of limited treatment response and poor response is heterogeneous and warrants attention when classifying a patient as such. Persistent DME in a post-hoc analyses of the DRCR.net and the VISTA/VIVID data was defined as a central subfield thickness (CST) of >250 µm for ≥6 months despite monthly treatment [14,15,25]. Other studies considered DME as refractory, when there was a less than 10% reduction in the CST after 3 monthly injections, a CST decrease <50 µm or a worsening of BCVA [26,27,28]. Another study considered a CST of ≥300 µm after 3 anti-VEGF injections in monthly intervals as unresponsive [29]. 

An expert panel recommended defining patients that did not show either a BCVA gain of >5 ETDRS letters and a reduction of ≤10% CRT or a CRT decrease of ≤20% after three consecutive anti-VEGF injections as therapy refractory to anti-VEGF [30]. A homogenous definition of therapy-refractive DME and poor treatment response is important in order to establish general guidelines on when and how to switch the treatment in DME. In some cases, the primary start with second-line treatments may be recommended based on patient characteristics and biomarker profiles. New drugs with different modes of action, such as the inhibition of the integrin pathway, are on the horizon [29], which further calls for clear and generally accepted definitions. The use of clinically available biomarkers will be fundamental to the optimal initial treatment choice, and algorithms on when to start treatment and how and when to switch are as well need.

## 5. Inflammatory and Glycemic Biomarkers in Diabetic Retinopathy (DR) and DME

Inflammatory and glycemic biomarkers are generally described in DR and are rarely specifically reported for DME. The Diabetes Control and Complications Trial showed that intensive glycemic control with reduction of A1c results in a 35–76% reduction in early stages of microvascular disease, including diabetic retinopathy [20]. C- reactive protein (CRP), an acute-phase protein widely used as a marker of inflammation, is increased in patients with DR compared to healthy subjects and increased in proliferative DR (PDR) compared to nonproliferative DR (NPDR) [30,31]. Advanced glycation end products (AGE) are the result of the non-enzymatic glycation of proteins and can be directly related to the pathology of diabetes [26]. Several AGEs, including N-epsilon-carboxymethyl lysine (N-Ɛ-CML) and pentosidine, are potential biomarkers for detecting early DR and for progression prediction [30]. Vascular endothelial growth factor (VEGF) is a vitreous biomarker for DR and plays a crucial role in the pathogenesis of DR through stimulation of angiogenesis, neovascularisation and vascular leakage [25,31,32,33,34,35,36]. Increased levels of platelet-derived growth-factor BB chain (PDGF-BB), which again induces expression of VEGF, are found in diabetic eyes [36]. Pigment epithelium-derived factor (PEDF) is an inhibitor of angiogenesis and is found in high concentrations in the vitreous humor of the healthy eye [37]. However, it is significantly decreased under hypoxic and inflammatory conditions such as in DR [18,33]. Ischemia in DR also stimulates erythropoietin (EPO) which in turn activates VEGF and contributes to neovascularization. EPO modulation has been shown to decrease VEGF-A expression by 48%, and it was hotly tipped as a novel treatment candidate for early DR also due to its neuroprotective effect on the retina [38,39]. 

In addition, Rho-Kinase/ROCK is an essential molecule in the pathogenesis of DME. The Rho kinase signaling pathways are upregulated in DME due to hypoxia and oxidative stress leading to vasoconstriction, endothelial impairment and leukostasis [40,41]. Initial pilot studies with intravitreal Rho-Kinase Inhibitors (+/−anti-VEGFF treatment) for the treatment of refractory DME have shown the first promising results [41]. Furthermore, several autoantibodies have been identified as serum biomarkers of diabetic complications [42]. Anti-hexokinase 1 antibody, for example, was detected as a novel serum autoantibody by using a combination of immunoblotting/immunoprecipitation and mass spectrometry. It is increased in the eyes with DR and DME [41,42,43,44,45,46].

## 6. Aqueous Humor Biomarkers and Their Impact on Treatment Response

Hyperglycaemia determined modifications in at least four major biochemical pathways: diacylglycerol (DAG)-protein kinase C (PKC), advanced glycation end products/receptor for advanced glycation end products, polyol (sorbitol), and hexosamine pathways [33,42]. The activation of those metabolic pathways leads to oxidative stress, inflammation, and vascular dysfunction, which results in the upregulation of cytokines and growth factors such as interleukins (ILs), angiopoietins, tumor necrosis factor, matrix metalloproteinases and VEGF, which can be assessed by aqueous humor analysis. In addition, markers of retinal glial cell and Mueller cell activation, like glial fibrillary acidic protein (GFAP), aquaporin 1 (AQP1) and aquaporin 4 (AQP4) can also be detected, appearing early prior to the onset of diabetic eye disease, and with levels rising with more severe stages of the disease [21,42,43]. All of these factors contribute to the breakdown of the blood–retinal barrier (BRB) and consecutive development of DME [8,41,42,43]. Given the complex pathology of DME driven by various pathways, one may assume that also the expression of angiogenetic and proinflammatory cytokines significantly differs among these patients. Reasonably, the response to treatment differs based on the pattern of present cytokines [21]. Furthermore, the chosen treatment will impact the cytokine profile under therapy. A patient with high levels of VEGF will nicely respond to drugs targeting exactly these proteins [22], while DME patients with higher levels of pro-inflammatory factors such as IL-8, IL-6, IL-1beta, etc., may benefit from alternative, anti-inflammatory treatment [39,40]. This can be actually seen in various studies assessing the cytokine levels using multiplex cytokine assay of patients in DME and monitoring their treatment response under different treatment regimens.

Recent published data, for example, show that baseline levels of VEGF in the aqueous humor using an enzyme-linked immunosorbent assay are significantly higher in anti-VEGF therapy responders compared to non-responders. DME patients, in contrast, who responded to corticosteroids but not to anti-VEGF agents showed significantly lower levels of VEGF than patients with rapid response [8,21,43,44,45,46,47,48,49]. Higher levels of angiogenetic factors, adhesion and inflammatory proteins, including ICAM-1, MCP-1, IL-6, IL-8, IP-10, IL-1b and VEGF, were detected both in the vitreous and aqueous humor of patients with DME compared to healthy controls [48,49]. The concentration of these factors positively correlates with macular thickness [8,47]. IL-6, a proinflammatory cytokine (and anti-inflammatory myokine), is secreted by macrophages. It stimulates the production of neutrophils, increases acute phase proteins and supports B-cell growth. In diabetes, IL-6 increases as DR stage advances [50]. Intercellular adhesion molecule 1 (ICAM-1) and the soluble form sICAM-1, a key intercellular adhesion molecule measured using solid-phase chemiluminescence immunoassay, is induced by IL-1 and TNFα and allows, e.g., leucocytes to bind to endothelial cells and evade into the tissue [51,52,53]. 

ICAM-1 has also been identified as an inflammatory biomarker in DME and correlates with the severity of macular edema and macular volume on OCT [22,46]. Intravitreal antiangiogenetic and anti-inflammatory treatments induce specific changes to the intraocular cytokine profile in DME. Under intravitreal triamcinoline (IVTA), for example, several factors including IL-6, IP-10, MCP-1, PDGF-AA, and VEGF have been reported to decrease, while treatment with the anti-VEGF antibody bevacizumab specifically affects the aqueous VEGF levels, but to a larger degree than IVTA [48]. Similar differences in their effects on the changes of aqueous humor protein levels were reported for the dexamethasone implant and ranibizumab: While the dexamethasone implant leads to a reduction of various adhesion and inflammatory proteins such as MCP-1, sICAM, sVCAM-1 and MIG, ranibizumab specifically reduces the VEGF and PlGF levels [49]. The concentration of proinflammatory cytokines such as IL-6 and IL-8 may even increase under anti-VEGF therapy in anti-VEGF refractory DME instances [44,54]. Other proteins, such as IL-8, MIF, TGFb1, TGFb2 and TGFb3, remained at similar concentrations, irrespective of treatment [44,49]. Obviously, some pathological cascades in DME are not targeted by currently available treatment options. The aforementioned data may explain why patients with chronic DME often present with high levels of pro-inflammatory cytokines. Correspondingly, chronic DME seems to show a favorable response to intravitreal steroids [49,51]. Additionally, aqueous humor analysis could be a useful tool for a targeted DME treatment based on the individual cytokine profile [54]. Further, new drugs with new modes of action will enter the market in the near future, broadening the applications of cytokine profiling for optimizing treatment choices.

## 7. Morphological OCT Biomarkers in DME

### 7.1. Optical Coherence Tomography (OCT)

In earlier days, fluorescein angiography (FA) was the only way to assess leakage and classify DME. Focal, petaloid, honeycomb and diffuse macular edema patterns were differentiated. FA still has its advances over OCT according to microaneurism/capillary leakage and assessing ischemia. Nowadays, non-invasive and simple-to-use OCT has emerged as the primary technique to detect and monitor DME. Depending on the balance between vascular leakage and fluid resorption, a discrepancy between the detection rates of macular edema (ME) in FA and OCT of about 44% has been reported with ME, which was visible on FA or on OCT, but not on both [30]. OCT quantifies central retinal thickness (CRT) and center point thickness, the main secondary outcome parameter in pivotal phase-3 trials in DME. It further identifies vitreoretinal interface abnormalities, including macular traction and vitreoretinal changes, intraretinal cysts, disorganization of inner retinal layers (DRIL), and hyperreflective foci, subfoveal fluid, and the integrity of the inner and outer retina and the photoreceptor layers. OCT is also used to localize edema to specific layers of the retina [55,56]. This imaging modality is widely used to monitor treatment response in DME in both clinical and research settings and is a highly valuable tool to identify and assess potential morphologic biomarkers.

### 7.2. OCT Pattern of DME 

**Figure 1:**
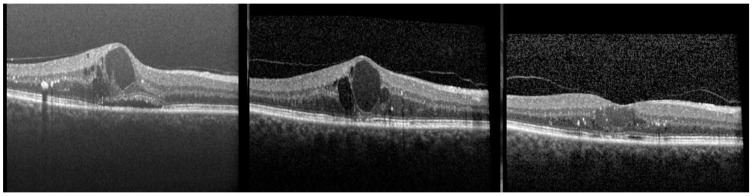
(**Left**) Patient with diabetic macular edema at baseline. The optical coherence tomography (OCT) exhibits hyperreflective foci, large cysts, subretinal fluid and a ganglion layer cyst. A vitreomacular adhesion is visible. Best-corrected visual acuity (BCVA) is 20/50. (**Middle**) OCT 10 days after the seventh ranibizumab injection on a monthly basis. Hyperreflective foci have significantly decreased, but large intraretinal cysts have remained, partly with homogenous hyperreflective content; some disruption of the ellipsoid zone (EZ) is visible. BCVA remained at 20/50. (**Right**) Four weeks after switching the patient to an intravitreal dexamethasone implant. Central retinal thickness and intraretinal cysts have significantly decreased. However, more hyperreflective foci are visible, the content of cystoid spaces is homogenously hyperreflective, and the EZ and the external limiting membrane is intact. BCVA has improved by one line to 20/40.

The mainly used differentiation of OCT DME pattern arises from the time-domain OCT (TD-OCT) era, where DME was differentiated into DME with CME, SRF (Figure 1), or into DME with sponge-like, diffuse retinal swelling [57].

This classification was used to explore the potential prognostic value of these different patterns and is nowadays also used to distinguish different subtypes of DME. The earlier TD-OCT devices had a low resolution, which impacts the classification. Whether IRF is perceived as cystic or diffuse is significantly influenced by OCT resolution and image quality and limits the interpretability of the results. Several authors claimed that diffuse macular edema is generally a poor prognostic factor for functional recovery [58,59,60]. Diffuse ME was linked to a greater inflammatory component [60]. Other authors suggested that diffuse DME is predictive of a favorable response to intravitreal steroids such as IVTA [61,62]. With the improvement of resolution and the advent of spectral-domain OCT (SD OCT), new and more advanced OCT classification systems have been suggested, aiming to distinguish DME based on its severity on its prognosis under therapy [63]. Unfortunately, these classification systems have not yet been extensively studied for their applicability to predicting treatment response. One classification system, called SAVE, was assessed under anti-VEGF (but not under intravitreal steroid) treatment and revealed that the parameter of focal leakage correlated with BCVA over the course of the treatment [64]. Another classification proposed to differentiate DME into four categories: vasogenic, nonvasogenic, tractional and mixed [65]. Both classifications have a limited predictive potential compared to the following morphological biomarkers in OCT. 

### 7.3. Intraretinal Cysts

Intraretinal cysts are the OCT landmark in DME. Cysts that represent intraretinal fluid accumulation can be distinguished on OCT by their location within retinal layers and by reflectivity and size. The location of the cyst can help to distinguish the underlying pathogenesis of macular edema [64,66,67,68,69,70,71,72,73]. Especially the deeply located microaneurysms in the deep capillary plexus contribute to the development of DME. They directly leak into the loose fibers of the Henle layer and lead to cyst development in the outer nuclear (ONL) and plexiform layer (OPL) [57,71,74]. Cysts in the inner nuclear layer (INL) (and consecutive ONL cyst development), on the other hand, result from a breakdown of the BRB with diffuse leakage from the superficial and deep capillary plexus triggered by proinflammatory cytokines. This pattern can also be appreciated in macular edema or inflammatory causes, such as pseudophakic ME or uveitic ME [57,69]. Considering the different nature of the cysts, it seems, therefore, reasonable that intraretinal cysts in the INL induced by general leakage of vessels are more responsive to either anti-VEGF or corticosteroids than fluid accumulation in the ONL, which mainly result from focally leaking microaneurysms [71]. Occasionally, cyst formation in the ganglion cell layer (GCL) can be found in DME (Figure 1). 

In contrast to the nuclear layers with their loosely connected tissue, the GCL has tightly connected tissue and is therefore not a great reservoir for fluid. It has remained presumptive whether GCL cysts are of exudative or degenerative nature. Not surprisingly, GCL cysts in DME correlate with slower visual recovery and a higher treatment demand for anti-VEGF injections than in DME patients lacking GCL cysts [72]. However, there are no data available on whether intravitreal steroids have a more favorable effect in these patients. The cyst size is another important parameter; the corresponding classification differentiates mild, moderate, and severe ME based on the size of cysts [56,73]. In this context, large cysts are defined as large foveal cystoid spaces with a horizontal width of ≥250 µm [71] (Figure 1). 

An early study proposed that large coalescent macrocysts in severe, long-standing DME may characterize retinal cystoid degeneration and are associated with Müller cell dysfunction and/or necrosis [74]. The presence of large cystic spaces correlates with greater central subfield thickness, higher prevalence of outer retinal damage, macular ischemia and with diffuse macular edema [75,76]. It is, therefore, not surprising that the sizes of ONL cysts are an important predictive marker for treatment response and functional improvements under anti-VEGF therapy [76]. Instead of measuring the cyst size, other authors took a different approach and assessed the remaining macular tissue between plexiform layers [77]. Both the remaining retinal tissue and large cysts are poor prognostic factors for visual recovery under treatment [75].

But what does the size of the cysts (or the remaining macular tissue) tell us about the optimal treatment choice? Previous studies proved that DME with large parafoveal cysts refractory to anti-VEGF treatment benefited from a switch to dexamethasone implant resulting in an improvement in BCVA [23,77,78,79]. Larger cysts also seem associated with longer disease duration and chronic DME. Chronic DME, in turn, favorably responds to intravitreal steroid treatments compared to anti-VEGF treatment [80]. This was confirmed in the FAME trials, where patients were treated with fluocinolone acetonide inserts; the chronic DME subgroup (≥3 years from diagnosis) had a greater proportion of patients with ≥15 letter increase than the acute DME subgroup [51,81]. 

To summarize, pathological pathways lead to different locations and the presentation of cysts within the different retinal layers. Large cysts with scarcely remaining retinal tissue are associated with chronic DME. The corresponding patients may benefit from intravitreal steroid treatment. 

### 7.4. Central Subfield Retinal Thickness (CSMT)

CSMT is still the main parameter to define and follow DME under treatment. Generally, a CRT > 250 µm in combination with retinal thickening is considered macular edema. In the case of foveal involvement, the term center-involving DME is used [82]. CRT was the primary outcome parameter in many phase II trials and the main secondary outcome parameter in pivotal phase III trials in DME [14,15,16,17,52,81,82,83,84,85]. CRT, central subfield thickness (CST), central foveal thickness (CFT), center point thickness (CPT), and central macular thickness (CMT) are all describing the mean retinal thickness of the central 1 mm on OCT and have been used to evaluate disease activity, progression and treatment response. However, CRT is only modestly correlated with baseline visual acuity (VA) or change in VA [84,85]; the correlation coefficients between CSMT and BCVA are low and further decrease with the duration of treatment [84]. 

Whereas immediate retinal thinning was accompanied by improvement of visual acuity under anti-VEGF therapy in the RIDE/RISE studies [83], other studies have shown that the proportion of patients with chronic persistent edema and increased CSMT gaining at least ten letters after 24 weeks was similar to patients with normal CRT and completely dry macula under (and irrespective of) various anti-VEGF agents [18,19]. BCVA can be severely decreased in the very thin atrophic retina, a long-standing DME with disorganization of the inner retinal layers (DRIL) and outer retinal degeneration, as well as in very thick retina due to a large amount of fluid. Thus, macular thickness is not a reliable prognostic or predictive marker for final functional outcomes. It is also not useful to predict the morphological treatment response of anti-VEGF vs. intravitreal steroid treatments. 

However, the initial decrease of CSMT can be used to predict treatment response in DME. A decrease in CSMT of ≥20% is often defined as a response that translates into a significant BCVA improvement of ≥10 letters, while a CSMT decrease of <20% is deemed as a poor response and is associated with limited functional improvement [86]. As larger intraretinal layer cysts in DME are generally associated with a greater CSMT, change in CSMT can also be used as indirect measure of treatment response to intravitreal steroids [87]. A greater macular volume and CRT have been linked to higher concentrations of ICAM-1 and sICAM-1; these proteins respond to intravitreal steroid treatment but not to anti-VEGF treatment [49]. On the other hand, VEGF levels were reported to correlate with CRT in DME, so CRT alone may not be an indicator of the optimal treatment choice [88]. A recent expert panel concluded that there is not enough evidence to assume a relationship between chronicity and inflammation of DME and the CRT [28].

To summarize, CRT and CMST alone may not be sufficiently strong parameters to guide treatment choices. Since a greater CRT is associated with large cysts, which is again a marker for chronic DME, intravitreal steroid treatment may generally be considered beneficial in advanced DME [89].

### 7.5. Disorganization of the Retinal Inner Layers (DRIL)

DRIL was defined as the horizontal extent in microns for which any boundaries between the ganglion cell–inner plexiform layer complex, inner nuclear layer, and outer plexiform layer could not be identified [90]. Disruption and disorganization have been hypothesized to result when bipolar axons snap after their elasticity limit has been exceeded due to edema [75]. DRIL is a prognostic biomarker that represents the disorganization or destruction of cells within the inner retinal layers, including bipolar, amacrine, or horizontal cells, and possibly indicates disruption of pathways that transmit visual information from the photoreceptors to the ganglion cells [90]. DRIL seems to be a prognostic biomarker for VA that is independent of CRT in eyes with baseline center-involved DME. Interestingly, DRIL is also associated with outer retinal damage of the ellipsoid zone (EZ) and external limiting membrane (ELM) [91]. Despite its degenerative origin, it may recover over time and under treatment. 

The resolution of DRIL under therapy is accompanied by functional improvement. This suggests that this biomarker has a prognostic value as an early marker for functional outcomes in therapeutic trials. Early recovery over three months in both DRIL and EZ parameters is a potential robust determinant of long-term VA recovery [92,93]. Therefore, DRIL change may represent a valuable and easily obtained noninvasive biomarker of VA that is applicable to clinical care and research studies [90]. The presence and persistence of DRIL under therapy indicate chronicity of DME, indicating that an early switch from anti-VEGF treatment to intravitreal steroids should be considered if DRIL does not improve properly. Hence, several studies proposed that dexamethasone implants may effectively ameliorate DRIL [93,94,95].

To summarize, DRIL represents the destruction of inner retinal layer cells, which may recover over time. Especially the short-term recovery of DRIL under treatment is an important predictor of functional vision gain. If DRIL persists under anti-VEGF treatment, an early switch to intravitreal steroids should be considered.

### 7.6. Disorganization of Retinal Outer Layer (DROL)

The integrity of the outer retina, the ELM, EZ, IZ, and retinal pigment epithelium (RPE) is beside DRIL, the most important prognostic parameter for visual recovery. The integrity of the EZ and ELM during treatment with either anti-VEGF or dexamethasone implant were directly correlated to long-term visual acuity [96]. Photoreceptor damage indicates chronic DME and is associated with macular ischemia [97]. Especially the ELM integrity seems crucial for the prediction of visual function recovery, which suggests a strong relationship with the photoreceptor cell status [98,99]. In case of outer retinal damage, even morphologically well-responding DME patients with complete resolution of fluid may fail to experience functional improvement. A recent study using machine learning (ML) to predict good and poor treatment responses after three monthly anti-VEGF injections (conbercept) revealed that the disruption ratio of the ELM at baseline was the third most important feature for the prediction of functional outcomes [79]. However, resolution and reduction of DME under anti-VEGF and steroid treatment may be accompanied by the restoration of the DROL [100,101]. In the case of refractive therapy DME with DROL under anti-VEGF therapy, some authors postulated that a switch to intravitreal steroids may be beneficial and lead to an improvement of DROL [102,103].

In the case of only limited recovery after the first application of intravitreal dexamethasone implants, however, the functional prognosis of these patients is rather poor [93]. It has to be highlighted, though, that the assessment of the outer layers is significantly impacted in severe DME, which may bias the interpretability of DROL and associated study outcomes. Shadowing and signal attenuation may not allow an accurate assessment of the outer retinal structures on OCT, leading to a false positive assessment. It is, therefore, not completely clear to what extent the morphological restoration of the hyperreflective ELM and EZ under therapy is true or whether the status of outer retinal integrity can simply be more reliably assessed after resolution and decrease of DME.

To summarize, DROL persisting after three months of treatment is, in general, a poor prognostic sign. When persistent DROL is observed under anti-VEGF treatment, the patient may benefit from a switch to intravitreal steroids. If early restoration is not encountered after the first administration of dexamethasone implant, the prognosis is rather poor.

### 7.7. Hyperreflective Foci (HRF)

HRF are defined as small discrete, well-circumscribed, dot-shaped lesions, 20–40 µm with equal or greater reflectivity than the RPE band on SD-OCT [104]. HRF can be found in various macular and retinal vascular diseases. Their exact entity is unknown. In DME, they were thought to represent lipid-laden macrophages suggesting inflammatory activity [105,106]. Another explanation linked HRF to activated resident microglial cells, which are initially present near ganglion cells and other inner retinal layers. This is in line with the fact that CD14 is increasingly found in the aqueous humor in the presence of HRF, which is released from activated microglial cells [60]. With the progression of diabetic retinopathy and DME and under the influence of inflammatory mediators, including VEGF, the inflammatory process spreads to the entire retina with outward migration of HRF, which are initially present in the inner retina to the outer retinal layers [104,107,108,109]. In the presence of HRF, not only CD14 but also Il-1β and IL-6 concentrations are increased, which underlines the inflammatory state in DME [8,60,110]. HRF are frequently found in the near vicinity of microaneurysms and intraretinal cysts [111], and not only at the retinal level but also in the choroid as a sign of advanced leakage activity [104,112,113,114,115,116,117,118]. The amount of HRF also represents a biomarker of disease severity in DR [109,110,111,112]. In general, a high amount of HRF seems to be a poor prognostic factor and is a very important predictive biomarker for DME (Figure 1). A higher amount of HRF was reported to result in a poor response to anti-VEGF therapy in several studies [79,113].

However, there were also studies that revealed the opposite, that a high number of HRF may well go along with satisfying treatment response to anti-VEGF [114]. In a large analysis of 712 DME patients, which aimed to predict the treatment response under anti-VEGF using machine learning, the sum of HRF at baseline was the most relevant feature, with an individual feature weight of 0.217 [117]. The second most important feature was the number of HRF in the ONL at baseline, with an individual relative feature weight of 0.135. In this paper, a good response was defined as a ≥50 µm reduction in CSMT after three monthly intravitreal injections with conbercept [117]. In another study, the treatment response was compared in treatment-naïve patients either started on ranibizumab or on dexamethasone implant [119]. In this study, dexamethasone implants showed superiority in reducing SRF and HRF compared to ranibizumab. Such was also observed in choroidal HRF [116]. Independently of a generally beneficial effect of intravitreal steroids on HRF, a large amount of HRF is also suggestive of a limited treatment effect of intravitreal dexamethasone implants, as a high number of HRF on SD-OCT was associated with early recurrence of DME after steroid implant [104]. Hyperreflective foci have been correlated to suspended scattering particles in motion (SSPiM) in OCT angiography [117]. SSPiM results from an extravascular OCTA signal. Due to the increase of particle efflux with optical properties after the breakdown of the BRB, these particles seem to induce a decorrelation signal caused by the Brownian motion of particles in intraretinal cysts [117,118]. In the presence of SSPiM in the ONL, DME eyes showed a reasonable response to anti-VEGF treatment, while SSPiM located in the INL seemed to better respond to intravitreal steroids [117].

In summary, HRF are, in general, a parameter suggestive of advanced DR and higher leakage activity in DME. Their presence is linked to inflammation and suggests high treatment need and shorter treatment effect, irrespective of the therapeutic strategy and the drug in use. The application of intravitreal steroids seems beneficial in cases with many HRF, namely in the outer retinal layers and the choroid, but more frequent and earlier recurrence of DME has to be expected. Overall, HRF seems to be one of the most important predictors for a stronger response to corticosteroids than to anti-VEGF drugs.

### 7.8. Hyper-Reflective Cystoid Walls

Apart from HRF also, hyperreflective cystoid walls have been studied as a potential predictive marker in DME. Some DME patients present with cystoid spaces that seem surrounded by a hyperreflective wall. The composition of these walls is unclear, but it was suggested that these walls could represent collagenous capsules [20,112,119]. Another explanation for that phenomenon might be gliotic tissue condensation surrounding the cystoid spaces [112,120]. Usually, they are found in association with HRF. Since hyperreflective foci seem to correspond to lipid-laden macrophages, inflammatory responses might contribute to the development of the hyperreflective walls and might indicate an increased inflammatory activity. It was shown that foveal cystoid spaces with hyperreflective walls persisted under anti-VEGF therapy, while cystoid abnormalities lacking this wall decreased under anti-VEGF treatment. Consistently, eyes with these features showed poorer VA and more severe EZ disruption compared to eyes with “normal” intraretinal cysts. Eyes with hyperreflective walls at baseline had a greater risk of persistent fluid after 18 months of continuous and PRN (pro re nata) anti-VEGF treatment [112]. While this parameter might be predictive of an unfavorable response to long-term anti-VEGF treatment, there are so far no data available that suggest that these patients may benefit from anti-inflammatory treatment. The association with HRF may indicate an inflammatory component. 

### 7.9. Central Choroidal Thickness (CCT)

CCT usually increases in the early stage of DR but decreases again when DR progresses [121]. The relevance of this finding, however, is unclear. CCT significantly declines in response to panretinal laser photocoagulation, possibly due to the VEGF downregulation, decrease in retinal and choroidal blood flow and subsequent atrophic changes [122,123,124]. CCT is thickened in the presence of DME, associated with both increased luminal and stromal structures and it seems related to the severity of DME [125]. In addition, the DME pattern impacts CCT as the thickest CCT might be associated with the presence of SRF [123]. A previous study assessed the choroidal thickness change after switching patients with persistent DME under ranibizumab treatment to either dexamethasone implant or aflibercept treatment. Switch to the dexamethasone implant and to aflibercept resulted both in a significant choroidal thinning and VA improvement compared to pre-treatment with ranibizumab [126]. A greater reduction in subfoveal CT, especially shortly after the application of a dexamethasone implant, was reported and may predict better anatomical and functional treatment response [127]. 

Another study found a greater CCT decrease after dexamethasone implant than after anti-VEGF therapy, resulting in better anatomical and functional outcomes. This decrease was associated with DROL reduction and SRF resolution [127]. The predictive potential of CCT in DME may thus be given, while robust data are lacking. The relevance of choroidal thickness as a predictive biomarker for treatment response has to be cautiously assessed as CCT response is impacted by various factors, and a possible correlation does not necessarily imply a causative effect. Nevertheless, choroidal thickness generally increases with the progression of DRP and increasing VEGF levels. Finally, CCT also increases in the presence of SRF, which consecutively results in a greater choroidal thickness decrease under steroid treatment [127]. In summary, in the absence of robust evidence, an independent role of CCT increase as a biomarker for disease severity remains to be determined, while CCT changes may also be associated with several other biomarkers of DR and DME severity.

### 7.10. Vitreomacular Interface (VMI)

The VMI is an important factor for treatment outcome and treatment needs in various retinal vascular and macular diseases [128,129] (Figure 1). Most studies that investigated the effect of the VMI on the resolution of DME are based on anti-VEGF treatment. There is evidence that posterior vitreous detachment (PVD) has a beneficial effect and leads to superior functional and morphological outcomes under anti-VEGF therapy [130]. A release of vitreomacular adherence (VMA) under anti-VEGF therapy was shown to be linked to a decrease in CRT [131]. One study with intravitreal dexamethasone-implant in DME found an association between the configuration of the VMI and the treatment response [132]. In contrast to current concepts, in this study, VMA and not PVD had a beneficial effect on functional treatment outcomes [132]. Regarding VMI, vitreomacular tractions that expert continuous mechanical traction on the fovea was correlated to worse prognosis with IVT treatment and therefore point to surgical add-on therapy (vitrectomy) [132,133,134]. In summary, the release of VMA had a positive treatment effect in DME under anti-VEGF, while limited evidence indicates that PVD in DME may limit the effect of dexamethasone therapy. Given the contradictory data on this biomarker, further data are warranted to understand the role of the VMI configuration in DME.

### 7.11. Subretinal Fluid (SRF)

Accumulation of SRF is thought to be either a sign of disruption of the external retinal blood barrier, secondary to damage in the tight junctions of RPE, or insufficient removal by an impaired RPE pump [34,38,62,63,135,136,137]. Intraretinal cysts, in contrast, arise from compromised tight junctions of the retinal vasculature and Muller cell dysfunction, which affect the water and potassium channels [136,137,138,139]. The presence of SRF has been associated with reduced retinal sensitivity [139]. SRF serves as a biomarker for functional and anatomic treatment response [23,52]. It is present in about 25–30% of patients with DME prior to treatment start [16] (Figure 1). However, there are a lot of conflicting results published on the impact of SRF in DME. Some studies did not find a correlation between SRF and the severity of DME [140,141]. Others found an association of SRF with higher concentrations of inflammatory cytokines, worse DME and a more chronic disease state [27]. 

There are studies reporting a favorable course of DME in the presence of SRF to anti-VEGF treatments [23,142,143], while others did not find a beneficial effect. In the RESTORE study, eyes with SRF at baseline had greater visual gain after one year [144]. Another study suggested that SRF may be an indicator of an earlier disease state and responds well to anti-VEGF if there are no other signs of chronicity of DME [145]. Other studies reported that SRF resolved rapidly under anti-VEGF therapy but reappeared when treatment was discontinued [146,147]. SRF may also be predictive of favorable steroid response compared to anti-VEGF [94,148]. The latter association seems reasonable as higher concentrations of inflammatory cytokines such as IL-6 and Il-8 have been reported, and, moreover, IL-6 continuously increases in poor anti-VEGF responders under VEGF therapy [22,149]. Another pro-inflammatory chemokine, MCP-1, has been correlated with SRF volume, and it decreases in patients who respond well to anti-VEGF therapy [22]. The reflectivity of SRF on OCT may be another indicator of potential treatment response. The reflectivity of the SRF on OCT seems to be correlated with the intravitreal VEGF levels in eyes with DME [149]. 

Although there is no specific staging of SRF reflectivity in DME, the reflectivity of intraretinal cysts has been divided into four categories: 1. low reflective, 2. heterogeneous reflective, 3. cystoid spaces with hyperreflective foci and 4. solid appearing cystoids. These different appearances were linked to the different content such as plasma, blood, hyaline or fibrinous material and macrophages, respectively—all of them are indicative of the severity of hyperpermeability [111,150].

In general, reflectivity may be a surrogate for the amount of lipids and proteins in the cystoid spaces: the higher the content of lipids and proteins, the higher the reflectivity. Usually, the reflectivity of SRF is lower than that found in intraretinal cystoid spaces in DME [111]. Considering that the reflectivity of cystic spaces is linked to increased inflammatory activity and severely impaired BRB, one may assume that this is also true for SRF. If so, steroids may be favored in the presence of highly reflective content. However, evidence is yet too weak to support the use of intravitreal steroids based on the OCT finding alone. Obviously, the predictive role of SRF and its reflectivity still remain to be determined. The presence of SRF probably has not a causative role but is a bystander phenomenon. 

It is also conceivable that the impact of SRF itself is not as strong, so its presence is to be considered in synergy with other biomarkers. It may also be that the presence of SRF in chronic and acute DME is different and linked to different states of the disease. Regarding SRF, it is really difficult to say if it is a prognostic factor. A decrease in VA is generally associated with subfoveal SRF in diabetic macular edema, but it seems to respond to IVT of either anti-VEGF or cortisone. It is not clear if it is really a prognostic or predictor factor. It could be just the result of the dysfunction of the outer retinal barrier in diabetic patients and therefore, the OCT images of SI/SE and apical junction line of the RPE could be the prognostic/predictor factor to be looked at in order to evaluate the risk of SRF and/or the response to treatments. 

To summarize, the impacts of SRF is not understood, given the heterogeneous findings published on this matter. The sparse papers which compared the drug efficacy and success based on the presence of SRF found a beneficial effect of steroids compared to anti-VEGF. However, large multicenter anti-VEGF trials in DME found that patients with SRF seem to have a favorable treatment response compared to patients without. While SRF in acute DME may be a predictor of a good anti-VEGF response, SRF in chronic DME may indicate the opposite. The reflectivity of SRF was correlated to VEGF levels. This indicates that VEGF plays a role in the development of SRF, while other studies linked proinflammatory proteins such as IL-6 and IL-8 to the presence of SRF.

## 8. Discussion and General Recommendation

This paper summarizes the known biomarkers in DME and their predictive potential for the effect of anti-VEGF and steroid treatments. A summary of so far detected biomarkers and their references are shown in Appendix A Table A1 and Table A2. 

Corticosteroids did not show an improved visual acuity compared to VEGF, neither in naive nor in persistent, chronic DME, but on an individual level, some patients may respond better to one, while others respond better to the other treatment approach, which is owed to the multifactorial origin of DME. A post hoc analysis of protocol I have shown that intravitreal steroids have the potential to lead to similar visual acuity improvements as anti-VEGF therapy, at least in pseudophakic eyes, where cataract progression does not impede functional performance [18,19,26,54,57,63,64,68,70,76,106,130,135,143,151]. However, they are associated with side effects, such as the development of cataracts and glaucoma, especially in predisposed patients [130,141,144,152]. Based on robust evidence that inflammatory proteins play a significant part in the pathophysiology of DME, corticosteroids represent an important option in our therapeutic armamentarium. Corticosteroids have a multifactorial mode of action and produce an anti-inflammatory effect through various mechanisms, including the decrease in the synthesis of inflammatory mediators and adhesion proteins as well as a decrease in VEGF levels [153]. Their use in the treatment of DME may be more comprehensive than anti-VEGF treatment, which targets only a part of the angiogenetic cascade [154]. 

Different choices of approved steroid treatments are available. Ozurdex, a 0.7 mg dexamethasone implant, was approved in 2014 in the US and Europe. According to the label, it is suitable for pseudophakic as well as phakic patients or patients insufficiently responsive to non-corticosteroid therapy. Iluvien, a 0.19 mg fluocinolone acetonide implant, was approved in 2014 in the US and parts of Europe. The US label supports the usage in patients who were previously treated with a course of corticosteroids without clinically significant elevation of IOP. The EU label recommends the usage in chronic DME is insufficiently responsive to other available therapies. According to the Euretina guidelines, steroids in DME are mainly second-line options, which should be restricted to anti-VEGF non-responders (after 3–6 injections, depending on the specific response of each patient). However, steroids are considered first-line therapy in patients with a history of major cardiovascular events and patients unwilling to come for monthly and frequent visits, respectively [51]. The dexamethasone implant as first-line therapy is namely recommended in patients with high-risk cardiovascular disease, poor compliance, severe edema (>500 μm), pseudophakic patients, patients scheduled to undergo cataract surgery, and patients with a history of vitrectomy [155]. Cost-benefit studies favor the dexamethasone or fluocinolone acetonide implant over other treatment options [156,157].

To decide, therefore, which DME treatment (laser (focal, subthreshold), anti-VEGF, corticosteroids, or vitrectomy) is the best option in individual DME cases according to international guidelines, recommendation depends on the baseline status of the patient, proper definitions, recognition of predictive biomarkers and identification of responsiveness to individual treatment [51]. Biomarkers in DME have been used to identify good and poor responders in general and to predict potential responses to different treatments. Higher CSMT at baseline, large intraretinal cystoid spaces, and more choroidal HRF are generally associated with limited treatment response but predictive of favorable treatment response to corticosteroids [64,95]. For other parameters, such as advanced ischemic maculopathy, the response of DME seems almost identical between anti-VEGF and dexamethasone [158].

Anti-VEGF agents are clinically effective in the majority of DME patients. However, a significant proportion (>40%) of patients with DME may not fully respond to this treatment [159,160,161,162,163]. Steroid implants have demonstrated good clinical outcomes with a favorable safety profile in treatment-naïve patients [162]. Pronounced changes in specific inflammatory parameters in the inner retina, such as reduction in HRF, DRIL extension, and CMT, are documented after steroid versus anti-VEGF treatment [164]. Early switching from anti-VEGF to steroids in insufficiently responsive patients has demonstrated favorable outcomes [152,163]. Compared with other steroids (triamcinolone, fluocinolone), intravitreal dexamethasone is associated with slightly fewer systemic side effects [155,165]. A rise in IOP (>25 mm Hg) was observed with intravitreal dexamethasone in only one-fifth of the injected eyes over a mean follow-up period of 16.8 months for all indications. IOP-lowering medication was required for 31% of the eyes [166]. Anti-VEGF treatment can also occasionally lead to spikes in IOP and to persistent OHT that requires IOP-lowering treatment [167]. Retinal ganglion cell layer thickness decreases in glaucoma and non-glaucoma patients treated with anti-VEGF agents and is associated with the number of injections [168].

The assessment of morphological biomarkers at baseline and over the course of treatment can help to guide the treatment strategy at treatment initiation and may help to decide for or against an early switch to steroid treatment. Circulating biomarkers and retinal imaging markers may permit more personalized treatment with better visual outcomes [169]. Besides the widely used OCT, also other image modalities may be helpful and contribute additional biomarkers. The quantification of short wavelength confocal 450 nm fundus autofluorescence, for example, may be considered as an imaging biomarker of retinal inflammation in DM, but this founding request further investigation of different fluorophores and their topographical distribution in the diabetic retina [170,171,172,173,174,175,176].

In the near future, machine learning approaches will further support individualized treatment and the best treatment choices based on a large scale of biomarkers and weighing of single feature relevance and interactions. DME patients with biomarkers suggesting a generally unfavorable treatment response to current treatment options may hopefully benefit from next-generation drugs currently in the pipeline, blocking, for example the integrin pathways or inhibiting tyrosine kinase. A timely switch to dexamethasone implant in order to avoid the irreversible loss of retinal cells due to persistent edema is essential for proper treatment and prevention of visual loss in patients with resistant and long-term DME.

## Data Availability

Not applicable.

## Appendix A

**Table A1:** Summary of biomarkers.

Biomarkers	Predictor of Good Response to Antivegf	Predictos of Good Response to Steroids	Candidate for First-Line Treatment with Steroids	Evidence *
INTRARETINAL CYSTS: -Large cysts ≥ 250 µm	NO	YES	YES	+++
DRIL	NO	YES	YES	++
HRF	NO	YES	YES	+++
HYPERREFLECTIVE CYSTOID WALLS	YES	NO	NO	+
VMA	YES	NO	NO	+
PVD	NO	YES	YES	+
SRF -acute DME	YES	NO	NO	+++
SRF -chronic DME	NO	YES	YES	+++
HIGH LEVELS OF VEGF IN AQUEOUS HUMOR	YES	NO	NO	++
HIGH LEVELS OF AQUEOUS OR SERUM: ICAM-1, MCP-1, IL6, IL8, IP-10, IL-1b	NO	YES	YES	+++

* Evidence was defined in several publications reporting on the respective biomarkers: **strong evidence** (+++) ≥ 4 published references in high-ranked journals; **moderate** (++) ≤ 3 published references in high-ranked journals; **weak** (+) = 1 published article in a high-ranked journal.

**Table A2:** Outlines of the references in respect to each individual biomarker.

Biomarker	References
Large Intraretinal Cysts	[68,69,70,71,72,73,74,75,76]
DRIL	[97,98,99]
HRF	[82,109,110,111,112,113,114,115,116,122]
SRF -acute DME	[23,146,147,150,151]
SRF -chronic DME	[22,98,115,152,154,177]
High Levels of Vegf in aqueous Humor	[35,48,123]
High Levels of Aqueous or Serum: ICAM-1, MCP-1, IL6, IL8, IP-10, IL-1b	[8,21,47,49,50,51,52,54]
